# Effects of Increasing the Nitrogen–Phosphorus Ratio on the Structure and Function of the Soil Microbial Community in the Yellow River Delta

**DOI:** 10.3390/microorganisms12122419

**Published:** 2024-11-25

**Authors:** Jinzhao Ma, Zehao Zhang, Jingkuan Sun, Tian Li, Zhanyong Fu, Rui Hu, Yao Zhang

**Affiliations:** 1 Shandong Key Laboratory of Eco-Environmental Science for the Yellow River Delta, Shandong University of Aeronautics, Binzhou 256603, China; mjz167448612@163.com (J.M.); zhangzh7717@163.com (Z.Z.); 912litian@163.com (T.L.); fzy721521@126.com (Z.F.); hurui1018@163.com (R.H.); zhangy8598@163.com (Y.Z.); 2 Institute of Restoration Ecology, China University of Mining and Technology-Beijing, Beijing 100083, China; 3 National Center of Technology Innovation for Comprehensive Utilization of Saline-Alkali Land, Dongying 257347, China

**Keywords:** nitrogen and phosphorus input ratio, bacterial community diversity, salt marshes, rhizosphere and bulk soil, Yellow River Delta

## Abstract

Nitrogen (N) deposition from human activities leads to an imbalance in the N and phosphorus (P) ratios of natural ecosystems, which has a series of negative impacts on ecosystems. In this study, we used 16s rRNA sequencing technology to investigate the effect of the N-P supply ratio on the bulk soil (BS) and rhizosphere soil (RS) bacterial community of halophytes in coastal wetlands through manipulated field experiments. The response of soil bacterial communities to changing N and P ratios was influenced by plants. The N:P ratio increased the α-diversity of the RS bacterial community and changed the structure of the BS bacterial community. P addition may increase the threshold, causing decreased α-diversity of the bacterial community. The co-occurrence network of the RS community is more complex, but it is more fragile than that of BS. The co-occurrence network in BS has more modules and fewer network hubs. The increased N:P ratio can increase chemoheterotrophy and denitrification processes in the RS bacterial community, while the N:P ratio can decrease the N-fixing processes and increase the nitration processes. The response of the BS and the RS bacterial community to the N:P ratio differed, as influenced by soil organic carbon (SOC) content in terms of diversity, community composition, mutualistic networks, and functional composition. This study demonstrates that the effect of the N:P ratio on soil bacterial community is different for plant roots and emphasizes the role of plant roots in shaping soil bacterial community during environmental change.

## 1. Introduction

N and P are the major limiting elements in terrestrial ecosystems and have received widespread attention. Previous studies have suggested that plant growth at high latitudes is usually limited by N, while at low latitudes, it is limited by P [1]. However, atmospheric N deposition continues to increase due to human activities, resulting in a shift from N-limited to P-limited plant growth at high latitudes [2,3]. The N input due to human activities increases more rapidly than the P input [4]. Unparallel N and P inputs may accelerate the bioavailability limitation of P. There are various sources of N, including the burning of fossil fuels, industrial emissions, and the abuse of N-rich fertilizers [5]. The P input has a single source, including mainly the weathering of rocks, which limits the amount of P input [6]. Although human activities have also increased the P content in soil and water, the overall increase in the P input is still too small in comparison with the N input [4]. The effects of the increased N:P ratio imbalance have been observed and reported in soil bacterial community structure and function, as well as in plant community species composition [4,7]. The impact of the continued increase in the N and P imbalance is being magnified. Currently, more and more studies are focusing on the effect of the N:P ratio [8].

Soil bacteria are considered to be important drivers in regulating biogeochemical cycles and the decomposition of plant litter and SOC in terrestrial ecosystems, and they play an important role in maintaining the structure and function of subsurface ecosystems [9]. Studies have shown the effects of N deposition on bacterial communities, and most of these studies found negative effects of N deposition on bacterial communities [10,11]. However, it was also found that N addition had positive effects on the bacterial community due to the fact that N addition increased carbon release from plant roots and alleviated carbon limitation during microbial growth [12]. P addition was found to mitigate the negative effects of N deposition on microbial communities, which is a process indirectly caused by P addition affecting soil carbon cycling and chemistry [13]. In contrast, in N-rich tropical rainforest areas, P addition had insignificant effects on bacterial community diversity but significantly changed the bacterial community composition [14]. However, there are fewer studies examining the effects of the N and P input ratio (due to N and P inputs) on bacterial communities and mostly focus on plant and soil C cycles. The response mechanism of the bacterial community structure and characterization to the imbalanced increase in the soil N:P ratio is still unclear.

In ecosystems where N and P are co-limited, plant productivity is usually determined by the N:P ratio [15]. The low level of the N:P ratio might not cause P limitation in plant growth [16], while at high levels of N input, the soil-available P (AP) content restricted plant growth [16]. Although N addition also induces phosphatase activity and thus increases P availability, the increase is insufficient to balance the increase in P demand [17]. Changes in the N:P ratio of terrestrial ecosystems directly affect plant biomass and indirectly affect consumers and decomposers [15]. For example, changes in nutrients can lead to shrinking root systems and reduced fine root biomass, thereby reducing bacterial community diversity [18]. Plants provide a habitat for soil microbial communities, so there may be consistency between the response of soil microbial communities and the response of plants to the N:P ratio [19]. However, there are differences in the physiology and growth strategies between plants and microorganisms, which may also lead to differences in their response to the N:P ratio [19].

The Yellow River Delta (YRD) is the most extensive and youngest coastal wetland [20]. The N:P ratio in the coastal wetlands of the YRD and Bohai Bay are severely imbalanced due to increased atmospheric N deposition, sewage discharge, and the continuous input of inorganic N and inorganic P pollutants from the Yellow River [8,21]. In YRD, studies of N:P ratios have focused on plant community structure and plant ecological stoichiometry [8,22] and mostly analyzed the relationship between soil C, N, and P contents, as well as their stoichiometric ratios and plant biomass, while the study of analyzing their effects on the soil microbial community from the perspective of the growing unbalanced degree of the N:P ratio has not yet been reported. In addition, previous studies have ignored the effects of plants on bacterial communities. In fact, the effects of N on the soil bacterial communities of different plants differ [23]. Under conditions of increased soil N:P imbalance, are there differences in the degree of variability in the N:P imbalance between RS and BS? Assuming there are, how will that affect the soil bacteria around them? What relationship exists among plant and soil N:P imbalances and microbial communities? To deepen our understanding of the effects of N and P ratios on coastal wetlands, an experiment with different N:P addition ratio gradients was set up in the coastal wetlands of the YRD to study (1) the response of soil bacterial communities to N:P input ratios and (2) the difference between the response of plant RS and BS bacterial communities to the N:P ratio by 16s rRNA sequencing from the perspective of α-diversity, community structure, and functional structure. We predicted that changes in the RS and BS bacterial communities are different when faced with changes in N:P ratios.

## 2. Materials and Methods

### 2.1. Description of Study Area

**Figure 1:**
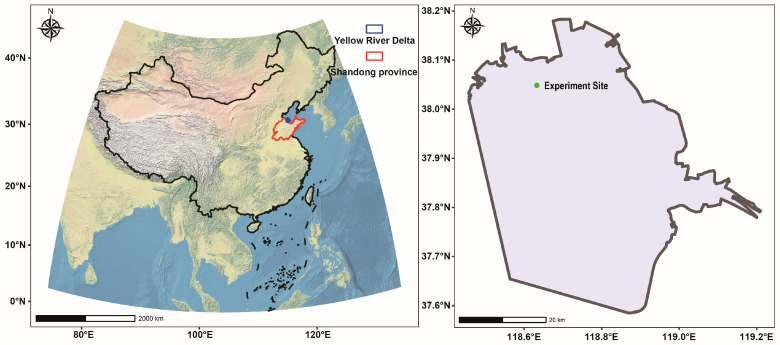
Location of the YRD and experimental site.

The present study was conducted in the YRD wetlands, Dongying, Shandong, China (E 118°44′2″, N 38°1′18″, Figure 1). The YRD enjoys a warm-temperate monsoon climate, with little climatic variation within its borders. The average annual sunshine hours are 2590–2830 h. The average annual temperature is 11.7–12.8 °C. The average annual precipitation is 530–630 mm, with 70% of the rainfall occurring in summer. The average annual evaporation is 1900–2400 mm. Influenced by its geographic location, the soil is dominated by coastal tidal saline soils. The soil type is mainly salinized tidal soil. The soils are sandy and clayey. More than 50% is salinized to varying degrees, and the degree of salinization is relatively high. Influenced by soil salinity, the plants are mainly halophytes, including *Suaeda salsa*, *Aeluropus sinensis* (*A. sinensis*), and *Limonium bicolor* as the main herbaceous plants; *Tamarix chinensis*, *Periploca sepium* Bunge and *Apocynum venetum* as the main shrubs; and *Robinia pseudoacacia*, *Fraxinus,* and *Populus* as the main trees.

In the YRD, the severe N deposition rate of 2.3 g/m^2^ has attracted the attention of scholars, and this rate is always increasing. However, the rate of increase in the P input does not keep up with the rate of increase in the N input [8]. Affected by N deposition, water and sediment regulation, and land use change, the soil N and P supply in this region is rapid [8].

### 2.2. Experiment Design and Soil Sample

The study area is about 120 m^2^, and twelve sample squares (1 m × 1 m) were randomly conducted in the study area. The minimum distance between each sample square was 2 m. The dominant plant in the study area is *A. sinensis*. Based on previous studies, we selected one CK (adding the same volume of distilled water as in treatment group) and three N:P ratio treatments. In the three N:P ratio treatments, P was added at 10 kg h^−1^, and N was added at 50, 150, and 450 kg h^−1^, respectively [8]. The N:P addition ratios were set to 5:1, 15:1 and 45:1, respectively. The three ratios corresponded to limited N, basic N:P ratio, and limited P conditions, respectively [24]. Urea and KH_2_PO_4_ were used for nutrient addition. Nutrients were added in May 2021. Soil sampling was conducted about 3 months after nutrient addition. BS was collected from 5–10 cm soil layer without plant growth. RS was collected from the soil nearer to the root (1–2 mm). Each soil was divided into two parts, one for microbial sequencing and the other for soil environmental factor determination.

### 2.3. Soil Environmental Factor Determination and Sequencing Analysis

The potassium dichromate volumetric method was employed to determine the SOC content. The total N (TN) and total C (TC) content were measured by an elemental analyzer (Vario macro cube, Elementar, Langenselbold, Germany). A pH meter and conductivity meter were employed to measure the soil pH and salt content, respectively. The molybdenum blue method was utilized to determine the total P (TP) and available P (AP) contents.

The total genome DNA of samples was extracted using the CTAB method. After dilution with sterile water, primers were used to amplify the V3 and V4 variable regions by PCR amplification. Then, the PCR products were sent to the sequencing company for high-throughput sequencing. More information about high-throughput sequencing is provided in Section S1 of the Supplementary Materials.

### 2.4. Statistical Analysis

The original DNA data were processed by the Qiime2. R (4.1.3) software vegan package (2.6-2), which was used to calculate the α-diversity of bacterial community. The formulas are as follows.

$$Shannon=-\sum_{i}^{n}pi\times \log_{2}pi$$

$$simpson=-\sum_{i}^{n}{pi}^{2}$$

where pi is the proportional abundance of species i. PICRUSt2 was used to predict the function of the bacterial community. The difference among treatments was tested using the LSD test by the agricolae package (1.3-5). Before performing the LSD test, the ANOVA test was first performed. Pearson correlation analysis was conducted to evaluate the correlations between soil physicochemical properties and bacterial community by the corrplot package (0.92). The ggplot2 package (3.3.6) was used for plotting.

The co-occurrence network was finished by the igraph package (1.3.4). There are 12 samples in each co-occurrence network. Before constructing the network, we removed OTUs that had low relative abundance (<0.01%) and disappeared in 4 samples. Connections with correlation coefficients less than 0.8 and p-values higher than 0.01 were filtered out by Spearman analysis. Finally, we used gephi software (0.9.2) for the visualization of the co-occurrence network.

With reference to [25], we performed Faprotax functional analysis based on OTU absolute abundance and annotation information. PICRUSt2 was used to analyze the KEGG pathway. Based on the KEGG Orthology, we have plotted the process of the N cycle [25].

## 3. Results

### 3.1. α-Diversity Analysis

**Figure 2:**
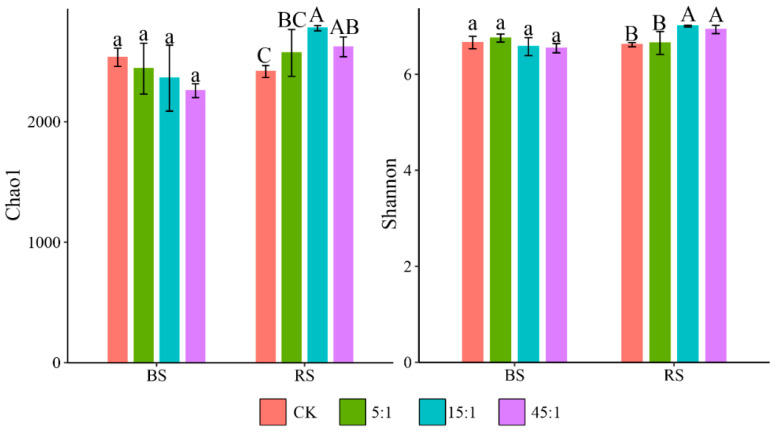
The Shannon and Chao1 indexes of bacterial communities in the RS and BS under different N:P ratio treatments. Different lowercase and uppercase letters indicate that the difference between N:P ratio treatments is significant at 0.05 level in the RS and BS, respectively. CK indicates the addition of distilled water.

The Shannon and Chao1 indexes of bacterial communities in the RS and BS of *A. sinensis* under different N:P ratio treatments are shown in Figure 2. The Shannon and Chao1 indexes of the BS bacterial community continued to decrease with increasing N:P supply ratios. The Chao1 and Shannon indexes were reduced by 10.92% and 1.76%, respectively, in the treatment with a high N:P ratio but showed an insignificant difference with CK. N and P addition increased the bacterial community diversity in the RS. Treatments with medium and high N:P ratios significantly increased the Chao1 index (by 14.89% and 8.48%, respectively) and the Shannon index (5.81% and 4.75%, respectively).

### 3.2. β-Diversity Analysis

**Figure 3:**
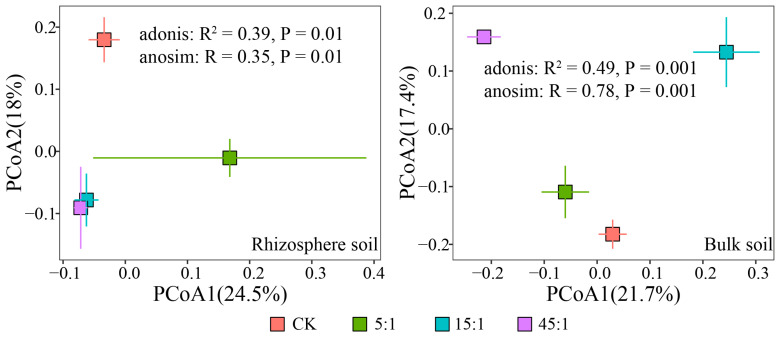
PCoA of bacterial communities in the rhizosphere soil and bulk soil. CK indicates the addition of distilled water.

Figure 3 shows the PCoA of bacterial communities at the OTUs level in the RS of *A. sinensis* and BS. The PCOA explained 42.5% and 39.1% of the variation in the RS and BS bacterial communities, respectively. ADONIS and ANOSIM analysis showed that N:P addition ratios had significant effects on the bacterial community of RS and BS (*p* < 0.01). The effect of the N:P ratio on the bacterial communities in RS was less than that of BS. In BS, the difference between N and P addition treatment and CK was gradually increased with increasing N:P supply ratio and had a significant linear relationship with the N:P ratio (Figure S1, *p* < 0.05).

### 3.3. The Dominant Phylum of the Bacterial Community

**Figure 4:**
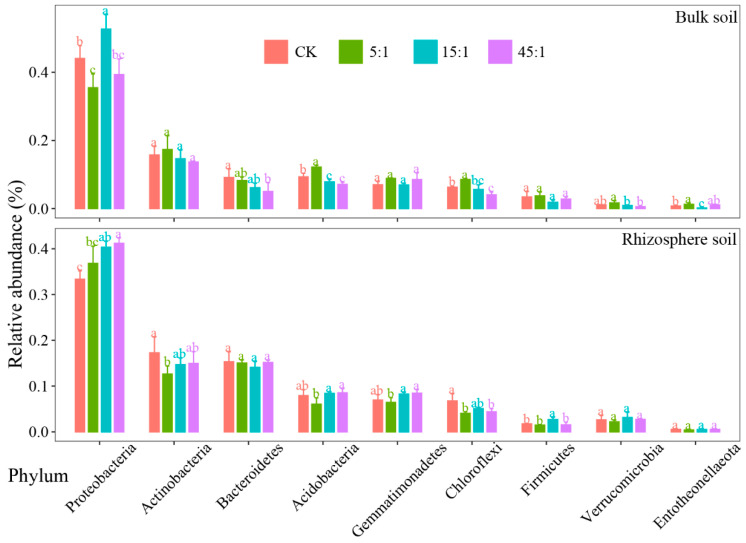
The dominant phylum of bacterial communities in the RS and BS communities. Different lowercase letters indicate that the difference is significant. CK indicates the addition of distilled water.

The highest relative abundance (Figure 4) was found in *Proteobacteria* (37.9%, 42.9%), followed by *Actinobacteria* (14.9% and 15.3%), *Bacteroidetes* (14.9%, 7.1%), *Acidobacteria* (7.7%, 8.1%), *Gemmatimonadetes* (7.5%, 7.8%), *Chloroflexi* (5.1%, 6.1%), *Firmicutes* (1.9%, 2.9%), *Verrucomicrobia* (2.7%, 1.1%), and *Entotheonellaeota* (0.5%, 0.8%). The high N:P ratio decreased the *Bacteroidetes*, *Acidobacteria*, and *Chloroflexi* of the BS by 44.8%, 23.7%, and 35.0%, respectively. The *Actinobacteria*, *Acidobacteria*, *Chloroflexi*, and *Verrucomicrobia* increased and then decreased with increasing N:P ratio, reaching a maximum at a low N:P ratio treatment. In RS, the *Proteobacteria* increased consistently with increasing N:P ratio and was significantly higher in the 15:1 and 45:1 treatments than in the CK (*p* < 0.05). Increasing N:P ratio decreased the relative abundance of the *Actinobacteria* and *Chloroflexi* of RS.

### 3.4. The Genus of the Bacterial Community

**Figure 5:**
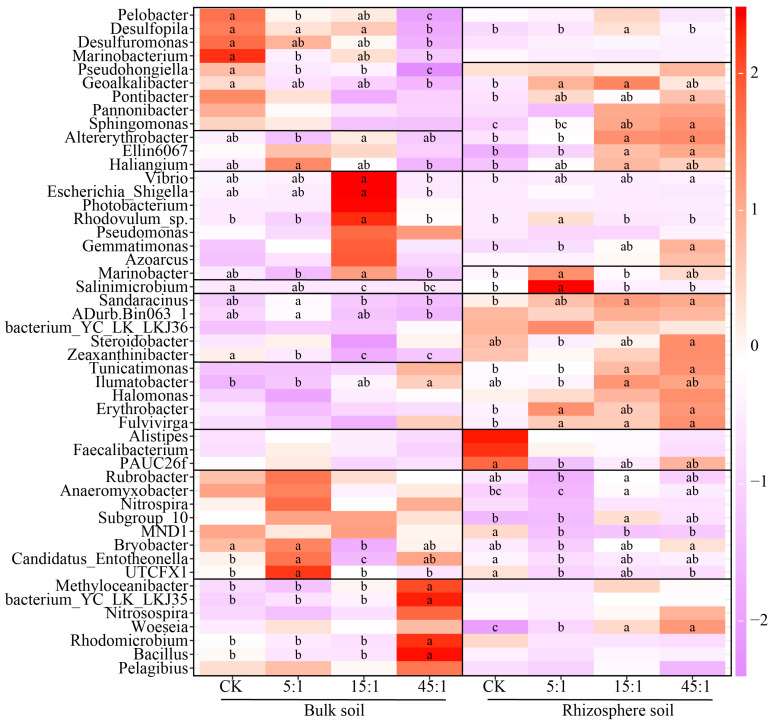
The dominant genera in the RS and BS communities. Different lowercase letters indicate that the difference is significant in the same soil. CK indicates the addition of distilled water.

The top 50 genera of the relative abundance of the RS and BS bacterial communities of the *A. sinensis* are shown in Figure 5. *Sandaracinus*, *bacterium_YC_LK_LKJ36*, *Steroidobacter*, *Zeaxanthiniabcter*, *Tunicatimonas*, *Ilumatobacter*, *Erythrobacter*, and *Fulvivirga* had higher relative abundance in the RS, while *Rubrobacter*, *Anearomyxobacter*, *Nitrospira*, *Subgroup_10*, *MND1*, *Bryobacter*, *UTCFX1*, and *Candidatus_Entotheonella* showed higher relative abundance in the BS. The high N:P ratio significantly increased *Bacillus*, *Rhodomicrobium*, *bacterium_YC_LK_LKJ35*, *Methyloceanibacter*, and *Ilumatobacter* (*p* < 0.05), and significantly decreased the relative abundance of *Pelobacter*, *Desulfopila*, *Desulfuromonas*, *Marinobacterium*, *Pseudohongiella*, and *Geoalkalibacter* in BS (*p* < 0.05). The relative abundance of *Geoalkalibacter*, *Pontibacter*, *Sphingomonas*, *Altererythrobacter*, *Ellin6067*, *Haliangium*, *Vibrio*, *Gemmatimonas*, *Sandaracinus*, *Tunicatimonas*, *Erythrobacter*, and *Fulvivirga* increased with the increasing N:P ratio in RS (*p* < 0.05). The increasing N:P ratio significantly decreased the relative abundance of *UTCFX1*, *Candidatus_Entotheonella*, *MND1*, and *PAUC26f* of RS (*p* < 0.05).

### 3.5. Bacterial Co-Occurrence Network

**Figure 6:**
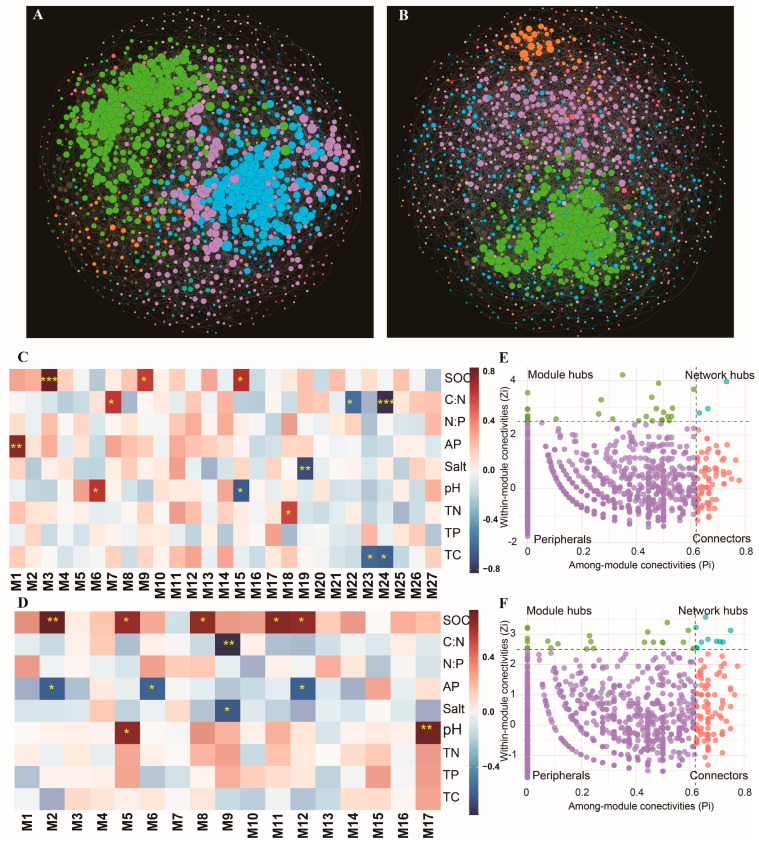
(**A**,**B**) Co-occurrence network of the RS and BS bacterial communities (12 samples). (**C**,**D**) Correlation between network modules and soil environmental factors in RS and BS. (**E**,**F**) Zi-Pi analysis of RS and BS. *, ** and *** indicate that the correlation coefficients are significant at the 0.05, 0.01, and 0.001 levels, respectively.

The numbers of nodes were 1284 and 1484, and the numbers of edges were 6502 and 6417 in the RS and BS, respectively (Figure 6A,B, Table S1). There are higher average degrees, network densities, clustering coefficients, and modules in the BS, while the network diameters and average path lengths are higher in the RS, suggesting that the BS has a more complex network. In addition, the BS network had a higher stability (Figure S2). The SOC content was significantly and positively correlated with several modules in both soils (Figure 6C,D). The AP content was negatively correlated with several modules in RS. Despite having more modules, only 3 network hubs were found in the BS network (Figure 6E), compared to 10 in the RS network (Figure 6F). Network hubs were rare species in both soils (Table S2). Among the 10 hubs in the RS network, there were 7 hubs belonging to the *Proteobacteria* and 2 hubs belonging to the *Bacteroidetes*.

### 3.6. Function Analysis

**Figure 7:**
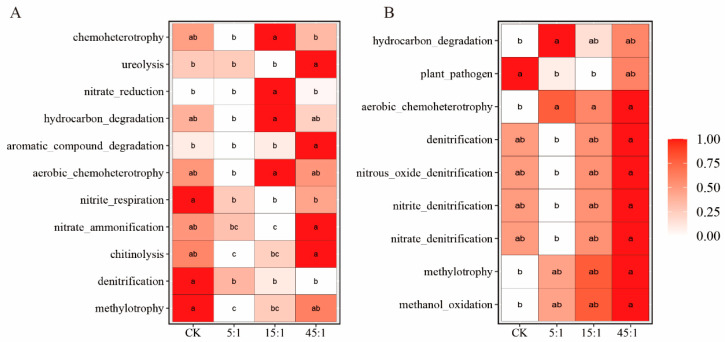
Faprotax functional analysis of the BS (**A**) and RS (**B**) bacterial communities. Different lowercase letters indicate that the difference is significant at a 0.05 level. CK indicates the addition of distilled water.

**Figure 8:**
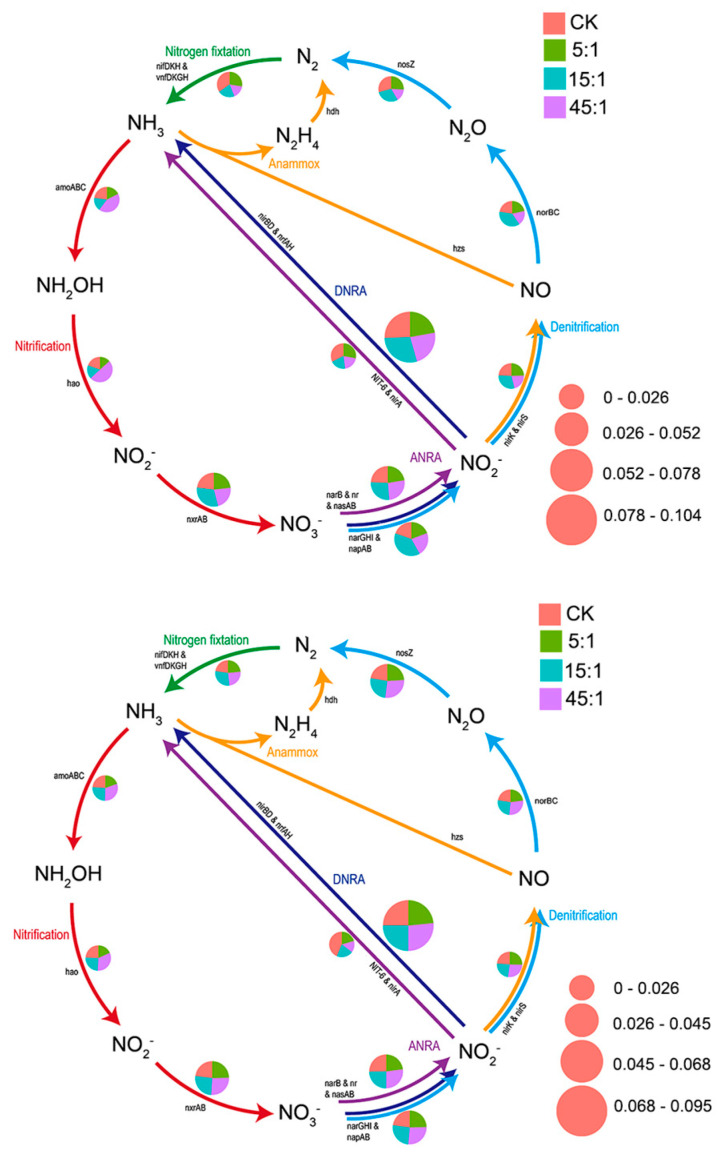
The pathway of N cycle of the BS (**top**) and RS (**bottom**) bacterial communities. CK indicates the addition of distilled water.

Differential functional compositions were observed between RS and BS (Figure 7, Table S3). In the BS, the treatment with a high N:P ratio enriched the urea decomposition, aromatic compound degradation, chitinolysis, and nitrification more, whereas the CK showed increased methylotrophy and nitrite respiration (Figure 7A). In the RS, more functions were associated with N metabolism. The treatment with a high N:P ratio increased microbial denitrification (Figure 7B). Increasing the N:P ratio reduced the abundance of plant pathogens but increased the abundance of aerobic chemoheterotrophs in RS. In the BS, increasing the N:P ratio enhanced the nitrification process, such as the process from NH_3_ to NO_3_^−^ (Figure 8, Table S4), but the abundance of N-fixing genes was decreased. In the RS, the high N:P ratio increased the abundance of denitrification genes (Figure 8). Additionally, functional predictions were made for 10 network hubs in the RS microbial network (Table S5). The results revealed four functions predicted, namely aerobic chemoheterotrophy, hydrocarbon degradation, iron respiration, and chemoheterotrophy. The chemoheterotrophy and aerobic chemoheterotrophy increased with the increasing N:P ratio (*p* < 0.05), while iron respiration exhibited the opposite trend (*p* < 0.05).

### 3.7. The Effect of Environmental Factors on Bacterial Community

**Figure 9:**
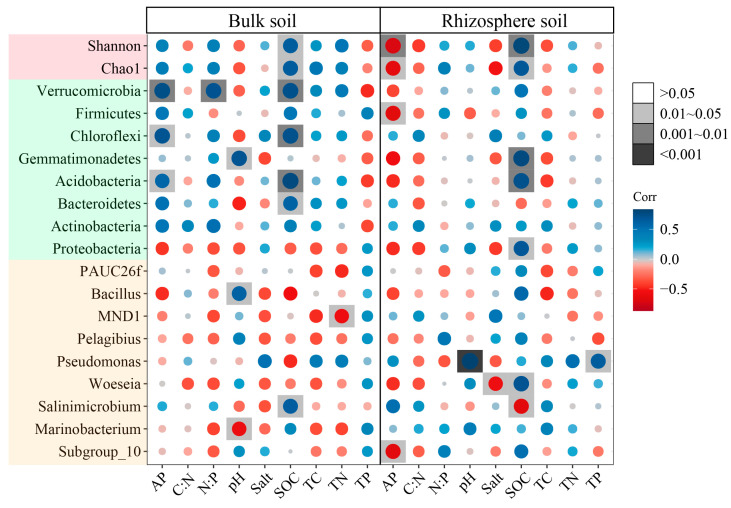
The correlation analysis between the physical and chemical properties and the bacterial community.

**Figure 10:**
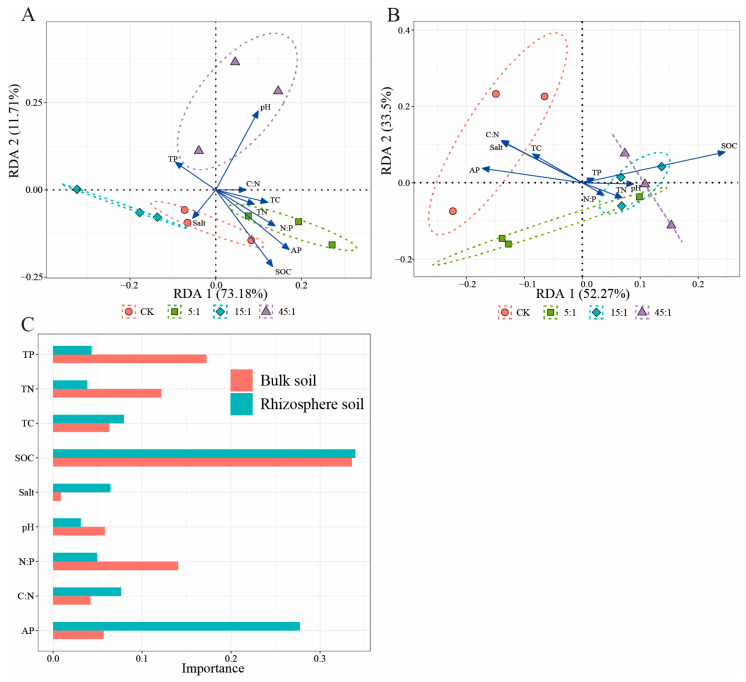
RDA analysis and multiple regression analysis. (**A**) RDA analysis for BS bacterial phlya. (**B**) RDA analysis for RS bacterial phlya. (**C**) Effect of the environmental factors on soil bacterial community Shannon diversity in BS and RS from multiple regression models. CK indicates the addition of distilled water.

The SOC and AP contents were most closely related to the bacterial community diversity of both RS and BS (Figure 9). In BS, the correlation coefficients of the SOC content with *Salinimicrobium*, *Bacteroidetes*, *Acidobacteria*, *Chloroflexih*, and *Verrucomicrobiad* were 0.63, 0.60, 0.77, 0.72, and 0.72 (*p* < 0.05), respectively. The AP content was significantly positively correlated with *Acidobacteria*, *Chloroflex*, and *Verrucomicrobia* (*p* < 0.05). In the RS, the SOC content was significantly and positively correlated with *Woeseia*, *Proteobacteria*, *Acidobacteria*, and *Gemmatimonadetes* with correlation coefficients of 0.70, 0.67, 0.72, and 0.77, respectively (*p* < 0.05). The AP content was negatively correlated with *Subgroup_10*, *Firmicutes*, Chao1, and Shannon in RS (*p* < 0.05). In both soils, the SOC content was positively correlated with the bacterial community α-diversity (*p* < 0.05). RDA analysis showed that the SOC content was the main environmental factor influencing the bacterial community phyla composition of BS and RS (Figure 10A,B). Except for the SOC content, the pH and AP content had significant effects on BS bacterial community’s phyla composition (Table S6). Similarly, the SOC content was the most key factor in the bacterial community diversity of both RS and BS (Figure 10C). In the RDA of both samples (Figure S3), the RS and BS bacterial community structures differed significantly, with AP explaining the most variation (R^2^ = 0.52, *p* = 0.001).

## 4. Discussion

In this study, N:P addition ratios increased the diversity of RS bacterial communities, which is inconsistent with most studies about the effect of N addition on bacterial communities. A previous study found that the N addition decreased the α-diversity of soil bacterial communities mainly by decreasing the soil pH [26]. In this study, a positive but insignificant correlation was found between the pH and α-diversity of the RS bacterial community. This may be due to the uptake of soil N by plants, which mitigates the effect of N addition on the soil [27]. In BS, the α-diversity decreased with increasing N:P ratio and was negatively correlated with pH, although insignificant, which demonstrated that the effects of N addition on the α-diversity of the RS and BS bacterial communities were different. On the one hand, P addition usually has insignificant effects on soil pH and is less likely to cause soil acidification [28]. In addition, the soils of the YRD are severely salinized and dominated by alkaline soils. And the present study was conducted in summer, when the BS evaporates severely and the pH does not easily decrease. On the other hand, P addition alleviated the P limitation of bacterial community under N addition and might have increased the threshold at which N addition induced a decrease in the bacterial community diversity [2]. Liu et al. [29] found that N input exceeding 32 g/m^2^ resulted in a decrease in the diversity of the RS bacterial community. In this study, there is a threshold between 15 g/m^2^ and 45 g/m^2^ of N addition that resulted in a change in the linear relationship between the RS bacterial community and the N addition gradient. In contrast, diversity was still increased compared to the CK, which further supports our previous conclusion that P addition may increase the threshold for the decrease in bacterial community diversity caused by N addition. Unfortunately, only three N:P addition gradient ratios were set in this study, and it was unable to further investigate the key turning threshold for decreasing the bacterial community diversity.

A negative correlation was found between α-diversity and AP content in the RS, but this phenomenon was not observed in the BS, which further indicates that nutrient addition has different effects on the RS and BS bacterial communities. The decrease in AP content was caused by an N-P imbalance that increased soil N availability, which in turn increased plant P demand. However, this may not be the main reason for the increased α-diversity of the RS bacterial community. In both soils, the bacterial community’s α-diversity showed a significant positive correlation with soil SOC content, which was similar to the findings of previous studies [30]. The N addition contributed to plants’ primary productivity [31], which in turn increased the root secretion release and apoplast production and alleviated microbial C limitation. In addition, the input of N and P contributed more to SOC than N addition alone [32]. N and P input can alleviate the N and P limitation in bacterial growth, thus reducing the N excavation and P excavation to SOC [33,34]. However, in BS, the continued utilization of bacterial communities can limit growth due to the absence of a stable C input.

The N:P ratio significantly altered the structure of the RS and BS bacterial communities, indicating that the bacterial community structures of both RS and BS were sensitive to changes in the N:P ratio. When under the treatment with a high N:P ratio, the most pronounced differentiation in bacterial community structure was observed, which is similar to the findings of the study by [35]. The higher N:P addition would promote great niche differentiation [35]. The bacterial community structure of BS is more sensitive to the N:P ratio than that of RS. More opportunities can be provided for different species that exhibit mutual adaptation to environmental stresses through such interactions [36].

There was a difference in the response of the dominant bacterial community phylum between the RS and BS to the N:P addition ratios, which was similar to the findings of previous studies [36]. The *Proteobacteria* had the highest abundance, which indicates its better adaptation to the environment [37,38]. The relative abundance of *Proteobacteria* in the RS bacterial community increased gradually with the increasing N:P ratio, while in the BS, the low- and high-N:P treatments were significantly lower than in the CK. *Proteobacteria*, *Actinobacteria*, and *Bacteroidetes* can grow rapidly in an environment with sufficient C content [39,40]. The N and P addition increased C release from plant roots, providing abundant C sources, which in turn promoted the growth of eutrophic taxa. The relative abundance of *Bacteroidetes* in BS decreased with increasing N:P ratios. The N and P addition alleviated the N limitation and P limitation during the growth of *Bacteroidetes*, but its growth was still C-limited. The relative abundance of the *Bacteroidetes* in RS was higher than in the BS, further indicating that plant root C sources limit the growth of eutrophic taxa. The medium and high N:P ratios significantly decreased the relative abundance of *Acidobacteria*, which is similar to the findings of a previous study [41]. The *Acidobacteria* belongs to the oligotrophic group [42], which mainly uses recalcitrant C in the soil. The nutrient addition may inhibit its ability to use recalcitrant C.

The relative abundance of *Ilumatobacter*, *Halomonas*, *Erythrobacter*, *Fulvivirga*, and *Haliangium* was significantly higher in the RS than in the BS. *Halomonas*, *Fulvivirga,* and *Ilumatobacter* were found to be plant-growth-promoting rhizobacteria. *Ilumatobacter* can help plants to suppress pathogenic bacteria and usually has a higher relative abundance in the RS [43]. *Halomonas* can increase soil permeability, improve soil structure, and provide nutrients to promote root and shoot growth and nutrient use efficiency [44]. *Haliangium* and *Erythrobacter* have the ability to decompose complex organic compounds [45]. The abundance of secretions in the RS promotes the growth of both. [29] found that the relative abundance of *Fulvivirga* was higher at low P effectiveness. In this study, the relative abundance of *Fulvivirga* increased with the increasing N:P ratio. *Fulvivirga* can promote the decomposition of organic P to satisfy the growth requirements of plants and itself. Higher levels of N input induce P limitation and promote the growth of *Fulvivirga* [29]. Wang et al. [30] found that vegetation cover reduced the relative abundance of *Rubrobacter*, which is consistent with the results of this study. *Anaeromyobacter* is an anaerobic bacterium, and its activity can be inhibited by root oxygenation [46]. The main function of *Nitrospira* is nitrification. On the one hand, root secretion inhibits nitrification, and on the other hand, nitrifying bacteria are less competitive for NH_4_^+^ compared to plant roots, which makes the relative abundance of *Nitrospira* lower in RS [47]. Our results showed that *Subgroup_10* was higher in BS, while Fu et al. [48](2022) found that *Sugroup_10* is the dominant genera using root secretion and has a higher relative abundance in the RS. We speculate that the salinity resulted in a higher relative abundance of *Subgroup_10* in the BS. *Subgroup_10* was found to be suitable for living in environments with higher salinity [49]. *A. sinensis* is a salt-secreting plant that can absorb salt from the soil and thus causes a decrease in soil salinity, which in turn changes the abundance of *Subgroup_10*.

In the BS, the relative abundance of *Bacillus* increased in the treatment with a high N:P ratio. *Bacillus* has good N-removal capacity and is therefore unaffected by increasing N levels [50]. In addition, *Bacillus* has the function of decomposing insoluble inorganic phosphorus minerals [51]. The P limitation of microorganisms also increased with the increasing N:P ratio; thus, the relative abundance of *Bacillus* with P-acquisition capacity increased. Sulfate-reducing bacteria (*Desulfuromonas*, *Desulfopila*) are important components that constitute nitrogen-fixing bacteria, and their abundance is affected by the N:P ratio, which is consistent with a previous study [52]. The relative abundance of *Sphingomonas* gradually increased with increasing N:P. *Sphingomonas* can decompose cellulose, alkanes, and aromatic hydrocarbons and can secrete carbohydrate hydrolases [53]. The increased N:P ratio caused an increase in root abscission and secretion in the RS, which promoted the growth of *Sphingomonas*.

The complexity of microbial co-occurrence networks is usually characterized by topological parameters, such as the number of nodes and edges [54]. We found that the co-occurrence network of the RS bacterial community has more nodes and edges, which suggests that there is higher diversity in the RS and more bacteria for coexistence. The enriched C of RS is suitable for the growth of microorganisms. Higher proportions of negative edges were found in BS, suggesting that BS bacteria are closer to a competitive relationship than a complementary relationship [55]. Soil nutrients are altered by the change in the N:P ratio. The bacteria better adapt to the environment and thus enhance their competitive advantage [56]. The bacterial community in the BS is more environmentally constrained than that in the RS [57]. Unlike RS, BS has no plant root system to transport the C source; thus, there is more competition for microbial available C. In RS, the selective effect of the altered N:P ratio on bacteria was mitigated by plants’ acquisition of nutrients. There were higher clustering coefficients and lower average path lengths in the BS co-occurrence network, suggesting closer communication between the nodes of the RS network. Microbial core groups are highly interconnected taxa in microbial communities, and changes in their abundance have important impacts on the structure and function [58]. More network hubs were found in the RS bacterial symbiotic network. The network hubs were all rare species, and most of them belonged to *Proteobacteria* and *Bacteroidetes*. It has been found that rare taxa of bacterial communities tend to be more closely linked to other members, occupy unique ecological niches, and are more sensitive to environmental changes [59,60]. Thus, although there are more nodes and edges in the RS bacterial co-occurrence network, its stability is more fragile compared to BS. We conducted the functional annotation for hubs and found that the functions were mainly focused on C utilization. The abundance of these functions was increased with the increasing N:P ratio. The abundance of several modules in the RS network was positively correlated with SOC content. Influenced by the N:P ratio, the release of organic matter from the plant root system increased, which enriched the microbially available C source [61].

Nutrient changes lead to changes in the soil microbial community structure, which in turn alters soil elemental cycling [55]. Nutrient inputs typically affect functional taxa involved in the C and N cycles. The increased N:P addition ratio increased the abundance of N-fixing genes and increased nitrification processing genes in BS, which is consistent with previous studies [62,63]. The abundance of denitrifying functional taxa increased in RS, along with the abundance of the chemoheterotrophy and aerobic chemoheterotrophy functions. In N-sufficient soils, the bacterial community increases the denitrification process as reactive N is released again during the decomposition of SOC content.

## 5. Conclusions

The N deposition caused by human activities has changed the N:P input ratio in terrestrial ecosystems. The Yellow River Delta, with its fragile habitats, is experiencing greater rates of N deposition and rapidly changing the N:P input–input ratio. In this study, we investigated the effect of N and P input ratios on bacterial communities in RS and BS. The N:P addition ratio significantly increased the community α-diversity of the plant RS community. Increasing the N:P ratio relieved N limitation and accelerated the process of soil nitrification in the BS and accelerated the denitrification process in the RS. The co-occurrence network of the RS bacterial community is more complex but has lower robustness compared to the BS. The response of the BS and RS bacterial communities to the N:P ratio differed, as influenced by SOC content in terms of diversity, community composition, mutualistic networks, and functional composition. This study illustrated that there are differences in the response of soil bacterial communities to N:P ratios as influenced by plant roots. This study emphasized that the interaction between environmental changes and plant root systems on soil microorganisms should be considered in future studies.

## Data Availability

The datasets presented in this study can be found in online repositories. The names of the repository/repositories and accession number can be found below: https://www.ncbi.nlm.nih.gov/, PRJNA949158 (accessed on 1 May 2020).

## Supplementary Materials

The following supporting information can be downloaded at: https://www.mdpi.com/article/10.3390/microorganisms12122419/s1, Figure S1. Linear regression of Bray-Curtis distance. BS, bulk soil. RS, rhizosphere soil. Figure S2. Network robustness analysis. Figure S3. RDA of all samples. Table S1. Basic topological properties of co-occurrence networks in bulk and rhizosphere soil. Table S2. The network hubs in bulk and rhizosphere soil bacterial community. Table S3. The function of bulk and rhizosphere soil bacterial community. The lowercase letter indicates that the difference is significant at 0.05 level. Table S4. The pathway of N cycle of bulk and rhizosphere soil bacterial community. The lowercase letter indicates that the difference is significant at 0.05 level. Table S5. Rhizosphere soil bacterial community network hubs. The lowercase letter indicates that the difference is significant at 0.05 level. Table S6. Monte Carlo test (per = 999) for top 10 phyla composition.
