# Supplementary material for: Effects of Increasing the Nitrogen–Phosphorus Ratio on the Structure and Function of the Soil Microbial Community in the Yellow River Delta
Source: Microorganisms. 2024 Nov 25;12(12):2419. doi: 10.3390/microorganisms12122419 (PMC11677714; doi:10.3390/microorganisms12122419)
Supplement: Supplementary file 1 [file microorganisms-12-02419-s001.zip › microorganisms-3300231-supplementary.pdf]

Supplementary material

Effects of increasing nitrogen-phosphorus ratio on the structure and function of soil microbial community in the Yellow River Delta

Jinzhao Ma [1], Zehao Zhang [1,2], Jingkuan Sun [1,3,*], Tian Li [1], Zhanyong Fu [1], Rui Hu [1] and Yao Zhang [1]

[1]*Shandong Key Laboratory of Eco-Environmental Science for the Yellow River Delta, Shandong University of Aeronautics, Binzhou, China.*

[2]*Institute of Restoration Ecology, China University of Mining and Technology-Beijing, Beijing, China*

[3]*National Center of Technology Innovation for Comprehensive Utilization of Saline-Alkali Land, Dongying, China*

* Correspondence: Jingkuan Sun

Email: sunjingkuan@126.com

Section S1:

Total genome DNA from samples was extracted using CTAB method. DNA concentration and purity was monitored on 1% agarose gels. According to the concentration, DNA was diluted to 1ng/μL using sterile water.

16S rRNA genes of distinct regions (16S V3-V4) were amplified used specific primer with the barcode. All PCR reactions were carried out with 15 μL of Phusion® High-Fidelity PCR Master Mix (New England Biolabs); 2 μM of forward and reverse primers, and about 10 ng template DNA. Thermal cycling consisted of initial denaturation at 98℃ for 1 min, followed by 30 cycles of denaturation at 98℃ for 10 s, annealing at 50℃ for 30 s, and elongation at 72℃ for 30 s. Finally 72℃ for 5 min

Mix same volume of 1XTAE buffer with PCR products and operate electrophoresis on 2% agarose gel for detection. PCR products was mixed in equidensity ratios. Then, mixture PCR products was purified with Qiagen Gel Extraction Kit (Qiagen, Germany).

Sequencing libraries were generated usingTruSeq® DNA PCR-Free Sample Preparation Kit (Illumina, USA) following manufacturer's recommendations and index codes were added. The library quality was assessed on the Qubit@ 2.0 Fluorometer (Thermo Scientific). At last, the library was sequenced on an Illumina NovaSeq platform and 250 bp paired-end reads were generated.

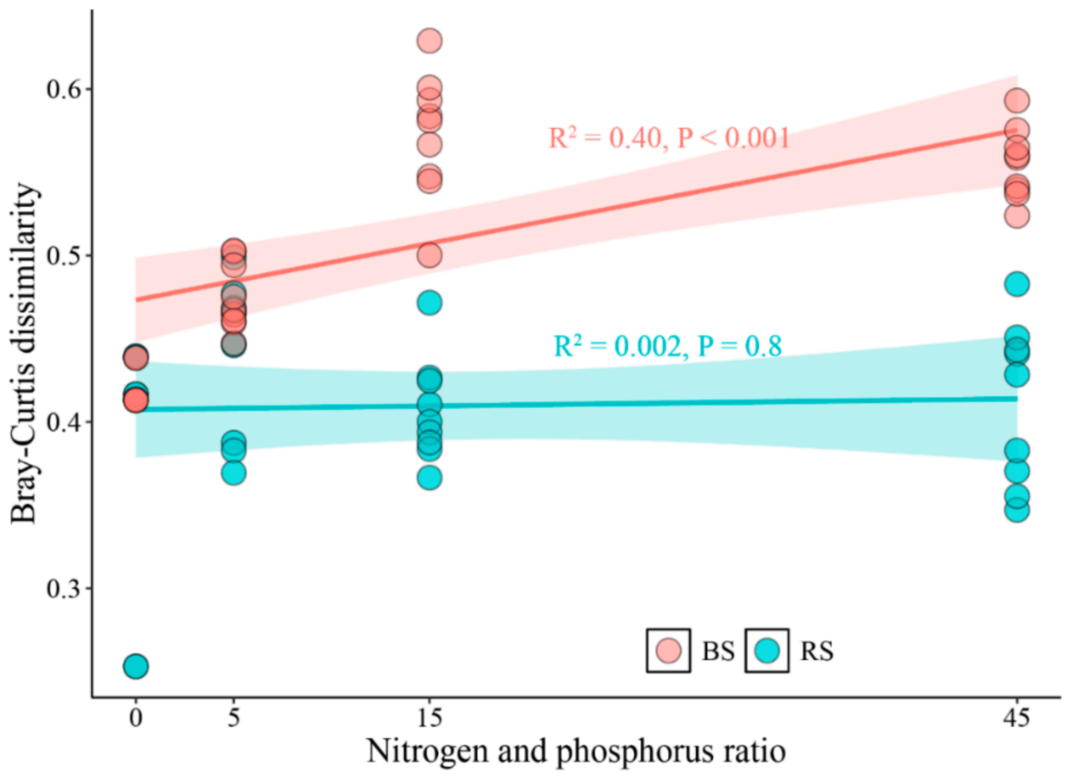

Figure S1. Linear regression of Bray-Curtis distance. BS, bulk soil. RS, rhizosphere soil.

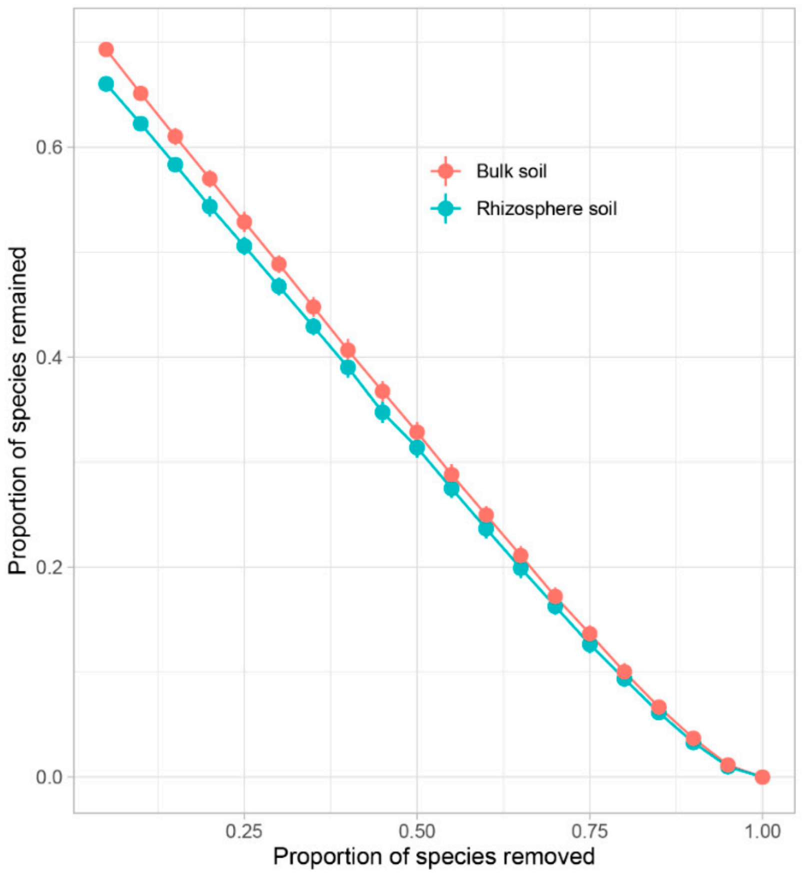

Figure S2. Network robustness analysis.

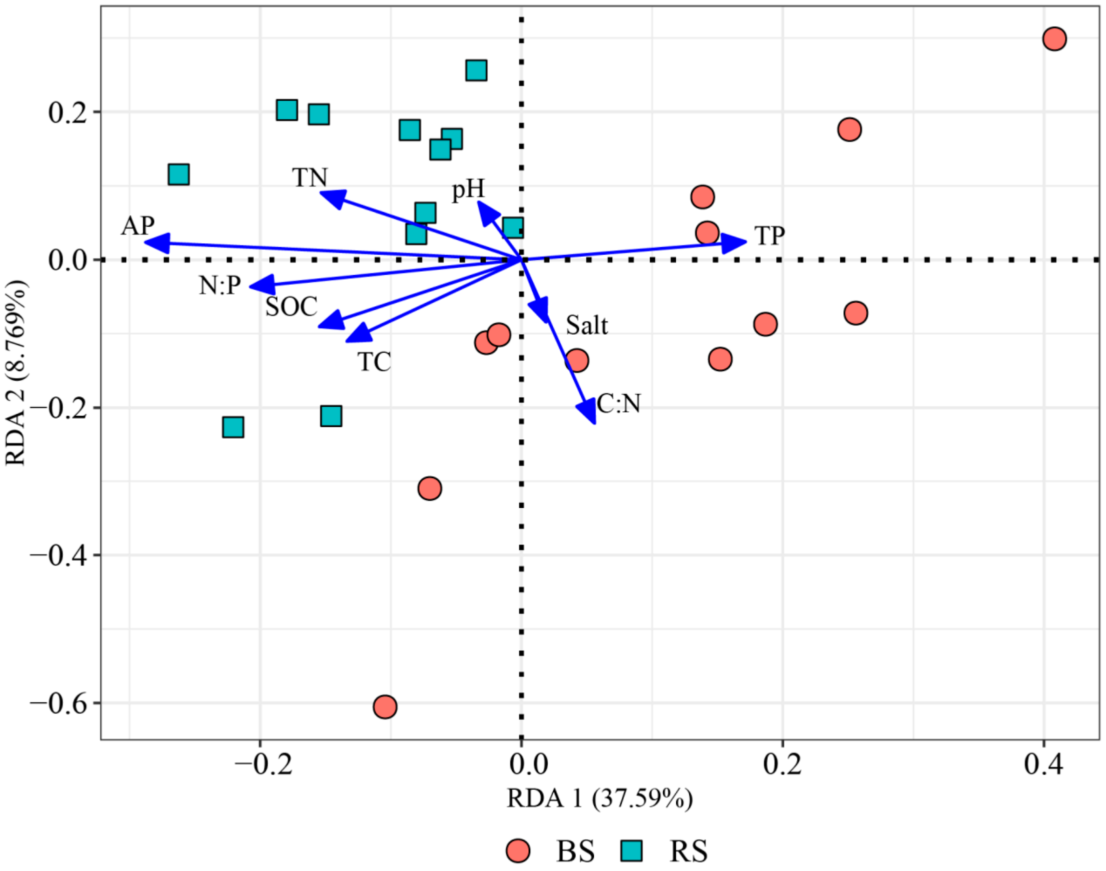

Figure S3. RDA of all samples.

Table S1. Basic topological properties of co-occurrence networks in bulk and rhizosphere soil.

Topological properties	Bulk soil	Rhizosphere soil
nodes	1284	1484
edges	6502	6417
positive edges	4196	4301
negative edges	2306	2116
average degree	10.127	8.648
average path length	4.676	5.145
network diameter	14	17
network density	0.0079	0.0058
clustering coefficient	0.323	0.318

Table S2. The network hubs in bulk and rhizosphere soil bacterial community.

Network	NO. OTU	Bulk soil relative abundance (%)	Rhizosphere soil relative abundance (%)
Rhizoshphere soil	OTU1	0.106	0.022
	OTU2	0.043	0.01
	OTU3	0.079	0.069
	OTU4	0.011	0.013
	OTU5	0.015	0.022
	OTU6	0.002	0.022
	OTU7	0.009	0.014
	OTU8	0.001	0.031
	OTU9	0.005	0.013
	OTU10	0.001	0.017
Bulk soil	OTU11	0.042	0.055
	OTU12	0.085	0.034
	OTU13	0.011	0.01

Table S3. The function of bulk and rhizosphere soil bacterial community. The lowercase letter indicates that the difference is significant at 0.05 level.

Soil	function	CK	5:1	15:1	45:1
Bulk soil	methylotrophy	0.068±0.007a	0.009±0.01c	0.024±0.018bc	0.046±0.01ab
	denitrification	0.138±0.017a	0.075±0.042b	0.049±0.035b	0.039±0.024b
	chitinolysis	0.205±0.042ab	0.111±0.014c	0.148±0.024bc	0.268±0.085a
	nitrate ammonification	0.072±0.049ab	0.046±0.035bc	0.007±0.002c	0.134±0.029a
	nitrite respiration	0.298±0.093a	0.121±0.057b	0.057±0.038b	0.164±0.013b
	aerobic chemoheterotrophy	9.368±1.04ab	6.099±0.201b	12.489±1.061a	9.381±3.456ab
	aromatic compound degradation	0.246±0.044b	0.15±0.035b	0.247±0.093b	1.164±0.339a
	hydrocarbon degradation	0.555±0.174ab	0.282±0.078b	0.991±0.578a	0.444±0.081ab
	nitrate reduction	1.292±0.241b	1.287±0.099b	3.125±1.181a	1.361±0.434b
	ureolysis	0.029±0.016b	0.028±0.022b	0.008±0.007b	0.088±0.041a
	chemoheterotrophy	11.965±1.235ab	8.929±1.52b	15.306±0.182a	11.158±3.452b
Rhizosphere soil	methanol oxidation	0.041±0b	0.061±0.027ab	0.076±0.018ab	0.085±0.026a
	methylotrophy	0.041±0b	0.061±0.027ab	0.076±0.018ab	0.085±0.026a
	nitrate denitrification	0.098±0.057ab	0.058±0.024b	0.102±0.036ab	0.138±0.019a
	nitrite denitrification	0.098±0.057ab	0.058±0.024b	0.102±0.036ab	0.138±0.019a
	nitrous oxide denitrification	0.098±0.057ab	0.058±0.024b	0.102±0.036ab	0.138±0.019a
	denitrification	0.098±0.057ab	0.058±0.024b	0.102±0.036ab	0.138±0.019a
	aerobic chemoheterotrophy	6.937±0.67b	8.763±0.776a	8.33±0.267a	9.257±0.926a
	plant pathogen	0.077±0.037a	0.024±0.022b	0.019±0.017b	0.054±0.027ab
	hydrocarbon degradation	0.72±0.065b	1.223±0.367a	0.802±0.148ab	1.022±0.347ab

Table S4. The pathway of N cycle of bulk and rhizosphere soil bacterial community. The lowercase letter indicates that the difference is significant at 0.05 level.

Soil	Pathway	CK	5:1	15:1	45:1
Bulk soil	narB or NR or nasAB	0.009±0.001ab	0.009±0.001b	0.01±0.001a	0.01±0ab
	norBC	0.002±0b	0.002±0b	0.004±0a	0.002±0b
	nosZ	0.007±0.001a	0.006±0a	0.007±0.001a	0.004±0.001b
	narGHI or napAB	0.008±0.001b	0.007±0b	0.015±0.003a	0.009±0.001b
	nirBD or nrfAH	0.027±0.002ab	0.023±0.001b	0.03±0.004a	0.024±0.002b
	nifKDH	0.004±0.001a	0.003±0ab	0.003±0.001bc	0.002±0c
Rhizosphere soil	narB or NR or nasAB	0.009±0.001ab	0.008±0b	0.009±0ab	0.01±0a
	norBC	0.002±0b	0.002±0b	0.002±0ab	0.003±0a
	nosZ	0.007±0.001b	0.008±0.001ab	0.009±0.001ab	0.009±0a
	nifKDH	0.003±0b	0.003±0b	0.003±0a	0.003±0b

Table S5. Rhizosphere soil bacterial community network hubs. The lowercase letter indicates that the difference is significant at 0.05 level.

Function	N0P0	N1P1	N2P1	N3P1
aerobic_chemoheterotrophy	0±0b	10.307±9.93ab	17.818±4.218a	21.455±10.326a
hydrocarbon_degradation	0±0b	3.145±5.447b	12.62±3.861a	7.765±7.156ab
iron_respiration	16.636±2.916a	4.62±5.38b	1.029±1.782b	1.431±2.479b
chemoheterotrophy	0±0b	10.307±9.93ab	17.818±4.218a	21.455±10.326a

Table S6. Monte Carlo test (per = 999) for top 10 phyla composition.

Soil	Rhizosphere soil		Bulk soil	
Factors	R^2	P	R^2	P
TC	0.11	0.58	0.14	0.49
TP	0.01	0.97	0.13	0.51
TN	0.05	0.8	0.08	0.65
pH	0.06	0.74	**0.52**	**0.03**
Salt	0.26	0.25	0.08	0.67
AP	0.26	0.24	**0.52**	**0.03**
N/P	0.02	0.92	0.26	0.25
C/N	0.26	0.25	0.04	0.81
SOC	**0.58**	**0.02**	**0.59**	**0.01**
